# Pattern of Repetitive Element Transcription Segregate Cell Lineages during the Embryogenesis of Sea Urchin *Strongylocentrotus purpuratus*

**DOI:** 10.3390/biomedicines9111736

**Published:** 2021-11-21

**Authors:** Nick Panyushev, Larisa Okorokova, Lavrentii Danilov, Leonid Adonin

**Affiliations:** 1 Bioinformatics Institute, 197342 St. Petersburg, Russia; nick.panyushev@gmail.com (N.P.); larisaok123@gmail.com (L.O.); 2 St. Petersburg State University, Department of Genetics and Biotechnology, 199034 St. Petersburg, Russia; lavrentydanilov@gmail.com; 3 Moscow Institute of Physics and Technology, 141700 Dolgoprudny, Russia; 4 Institute of Biomedical Chemistry, Group of Mechanisms for Nanosystems Targeted Delivery, 119121 Moscow, Russia; 5 Institute of Environmental and Agricultural Biology (X-BIO), Tyumen State University, 625003 Tyumen, Russia

**Keywords:** repetitive DNA, noncoding RNA, mobile elements, repetitive elements (REs), transposable elements (transposons; TEs), sea urchin *Strongylocentrotus purpuratus*, embryogenesis, gastrulation, primary mesenchyme cells (PMCs), scRNA-seq

## Abstract

Repetitive elements (REs) occupy a significant part of eukaryotic genomes and are shown to play diverse roles in genome regulation. During embryogenesis of the sea urchin, a large number of REs are expressed, but the role of these elements in the regulation of biological processes remains unknown. The aim of this study was to identify the RE expression at different stages of embryogenesis. REs occupied 44% of genomic DNA of *Strongylocentrotus purpuratus*. The most prevalent among these elements were the unknown elements—in total, they contributed 78.5% of REs (35% in total genome occupancy). It was revealed that the transcription pattern of genes and REs changes significantly during gastrulation. Using the *de novo* transcriptome assembly, we showed that the expression of RE is independent of its copy number in the genome. We also identified copies that are expressed. Only active RE copies were used for mapping and quantification of RE expression in the single-cell RNA sequencing data. REs expression was observed in all cell lineages and they were detected as population markers. Moreover, the primary mesenchyme cell (PMC) line had the greatest diversity of REs among the markers. Our data suggest a role for RE in the organization of developmental domains during the sea urchin embryogenesis at the single-cell resolution level.

## 1. Introduction

Eukaryotic genomes are larger and more complex than those of prokaryotes, and they also significantly differ in size. However, there is no correlation between the genome size and the organism complexity. For example, the genome size of some amphibians, bony fish, and some arthropods exceeds by twice or more the size of the human genome, however, we can hardly claim the same order of complexity for these organisms [1,2,3,4]. In an in-depth analysis of sequences of eukaryotic genomes, it became clear that the differences in genome size are due to the presence of different amounts of repetitive DNA, while the number of functional genes varies within the same range (up to 25,000) [5]. This phenomenon was described by Waring and Britten a half-century ago using reassociation studies [6,7]. The presence of repetitive DNA is a distinctive feature of eukaryotic genomes; it occupies a major part of the overall nuclear DNA.

All repetitive DNA in the genome is grouped into two large classes according to the genomic organization: tandem repeats and dispersed repeats or transposable elements (transposons, TE) [8,9]. Although much progress has been achieved in understanding the role repetitive elements (REs) play in a host genome, we are still far from having a comprehensive picture of the delicate evolutionary interplay between a host genome and the RE. The biological role of repetitive DNA is not fully understood even though it is of great importance for understanding the basic matrix processes in the norm and in pathology. Therefore, numerous studies are currently focused on the evolutionary role of RE in gene regulation mechanics. At present, TEs remain the most studied from this point of view [10,11,12,13,14].

It is known that most of the mammalian and other studied eukaryotic genomes are transcribed during normal embryonic development [5,15]. The same is true for genetic disorders that lead to the development of diseases in humans (and other multicellular organisms) [16]. Surprisingly, about 85% of the human genome is transcribed into primary and processed transcripts [17]. As a result of the wide transcription of almost the entire genome sequence, different RNA types are synthesized: protein-coding RNA (or mRNA) and several classes of non-coding RNA (ncRNA) [18,19]. Growing evidence shows that most ncRNA transcripts are derived from genomic mobile elements [17]. At present, the functions of most ncRNA transcripts are unknown. Meanwhile, recent studies of individual members of different classes of noncoding RNAs show increasing evidence that they play an important role in epigenetic regulation [20].

A deeper understanding of RE function has been limited by difficulties in analyzing the RE transcription in RNA-seq data. Analysis of RE transcription is challenging for short-read RNA sequencing due to their highly similar sequences and repetitive nature. RNA-seq reads that originate from one locus can be ambiguously aligned to many TEs sharing similar sequences dispersed throughout the genome. Due to these barriers, conventional RNA-seq analyses of REs have either discarded multi-mapping alignments [11] or combined TE expression to the subfamily level [21,22,23]. Pseudoalignment approaches take up the challenge and perform well, which is confirmed by comparing results with qPCR [24].

Moreover, the analysis of RE expression in scRNA-seq data becomes even more complex. Several studies have made decent attempts to analyze RE expression at the level of single cells. For example, RE expression at the single-cell resolution was detected in *Drosophila melanogaster* brain cells. The authors looked at chimeric transcripts containing transposons and mRNA and concluded that transposon expression was driven by neighboring genes, and this diversified the neural transcriptome [25]. TE expression analysis among different cell types of the *Caenorhabditis elegans* early embryo have shown that LTR elements are mostly expressed in undifferentiated cells and might play a role in pluripotency maintenance and activation of the innate immune response. Meanwhile, non-LTR are expressed in differentiated cells, in particular in neurons and nervous system-associated tissues, and DNA TE are homogenously expressed throughout the *C. elegans* early embryo development [26]. It should be emphasized that these works used alignment with the Burrows algorithm. Traiber et al. point out the functional significance of chimeric transcripts. At the same time, Ansaloni et al. warn that these chimeric transcripts can significantly over-estimate RE expression levels.

He and co-authors managed to capture specific RE types that were expressed in subpopulations of human embryonic stem cells and were dynamically regulated during pluripotency reprogramming, differentiation, and embryogenesis. In addition, RE expression was detected in somatic cells, including human disease-specific REs that are undetectable in bulk analyses [27]. A construction of RE-containing ncRNA references using bulk RNA-seq data was used by Shao [28]. This strategy was applied to mouse embryonic stem cells and successfully captured the expression profile of endogenous retroviruses in single cells. The authors managed to show the dynamic TE expression at preimplantation stages and revealed 146 TE-containing ncRNA transcripts with substantial tissue specificity during gastrulation and early organogenesis [28].

Experimental research on human embryonic development is complicated by ethical issues and the impossibility of *in vitro* cultivation of an embryo for more than two weeks according to the “14-Day Rule”. In our opinion, the purple sea urchin *Strongylocentrotus purpuratus* is a convenient model for studying epigenetic regulation based on noncoding RNAs. Sea urchins have been used by biologists as a model organism for over 150 years [29]. In addition to the close phylogenetic position of the sea urchin to chordates, this object has a number of features that make it convenient to work with [29,30]. Therefore, the data obtained from using this object can be extrapolated to mammals, including humans, with greater probability and accuracy.

Using this model organism, Davidson and colleagues were able to present the first experimental validation and systematic description of the gene regulatory network that controls the specification of endoderm and mesoderm for sea urchin embryogenesis [31]. The network contained more than 40 genes and was the first step to studying its complex multilevel organization. After the Purple Sea Urchin genome was sequenced, and due to availability of high-throughput gene expression data, researchers throughout the world were able to add to and expand the knowledge of the developmental gene regulatory network [32,33,34]. Thus, years of morphological studies of sea urchin embryos have been supported by molecular genetic data, allowing researchers to clearly distinguish the trajectories of differentiation of each blastomere of the growing embryo.

The evidence concerning gene regulatory network (GRN) in single-cell resolution is only starting to emerge, and none of the existing experiments have been made while taking into account the highly repetitive nature of deuterostomes’ genomes. In this study, we refined the repertoire of the REs of the latest *S. purpuratus* genome build. Using bulk transcriptome data at different timepoints, we revealed the transcription pattern of those REs from the unfertilized egg to the late gastrula stage. Considering the bulk RNA-seq-derived data on the activity of each of the RE instances and scRNA-seq data, we also discovered several RE-derived transcripts that are important for the formation of main developmental domains of the sea urchin embryo. This data may be later used to identify new mechanisms of ncRNA-dependent modulation of transcription during the embryogenesis of sea urchin and other deuterostomes.

## 2. Materials and Methods

### 2.1. Data

The publicly available transcriptome data from *S. purpuratus* embryos were downloaded from the SRA database under accession number PRJNA531297. The data contained RNA-sequencing reads of unfertilized eggs, embryos from 2, 4, 8, 18, 32, 64, and 128 cell stage (blastula stage) and 24, 40, 41, and 48 h after fertilization (gastrula stage), which were maintained at 15 °C.

Raw reads of the single-cell RNA-sequencing data were downloaded from the SRA database stored under accession number GSE149221 [35]. The dataset contained eight developmental stages of *S. purpuratus*, from the eight-cell stage to late gastrula stage.

### 2.2. RE Annotation and Classification

Repetitive elements in *S. purpuratus* genome version 5.0 were identified and classified by RepeatModeler 2.0.1 using the Dfam 3.1 database. Consensus sequences of repetitive elements (consensi.fa output file) were used for downstream analysis of differentially expressed genes. The RepeatMasker was used for locating the discovered REs in the genome.

### 2.3. Bulk RNA-Seq Analysis

For the RNA-seq data, gene expression abundances were computed using the Kallisto software (0.46.0) with accompanying sleuth R package (v0.30.0). Transcriptome reference files for pseudoaligment were prepared by concatenating reference transcriptome (GCF_000002235.5_Spur_5.0_rna.fna) of protein-coding genes with consensus sequences of repetitive elements, identified by RepeatModeler (consensi.fa output file).

### 2.4. Defining the Transcriptionally Active RE Instances

Identification of transcriptionally active RE instances was performed using TEcandidates v.2.0.5., which assesses the active RE instances based on de novo transcriptome assembly with Trinity. TEcandidates takes as input the RNA-seq data (fastq.gz), the genome sequence (Spur_5.0.fa), and the RE annotation file (Spur_5.0_genome.fna.out.gff). RE annotation file was produced by RepeatModeler and contained coordinates of all RE copies. TEcandidates returns a list of coordinates of the candidate REs being expressed, the REs that have been removed, and the genome sequence with removed REs as masked. This masked genome is suited to include REs in downstream expression analysis, as the ambiguity of reads coming from REs is significantly reduced in the mapping step of the analysis. We used the list of expressed RE for constructing reference transcriptome for the further steps. The RE instance was included in the resulting annotation only if it had been expressed in at least one embryo stage.

### 2.5. Analysis of scRNA-Seq Data

Considering the repetitive nature of non-coding DNA, we chose the pseudoalignment approach with expectation-maximization RE resolution. This approach was implemented in the Kallisto Bustools suite and was used for single-cell primary analysis [36]. The strategy for preparation of the reference transcriptome was different from the one used for bulk RNA-seq analysis. In contrast to the use of consensus RE sequences, we used locus-specific sequences of transcriptionally active RE. For this purpose, we extracted the sequences included in the list of expressed REs obtained by TEcandidates from the Spur_5.0 genome. Next, we concatenated the resulting fasta file with the transcriptome fasta file (GCF_000002235.5_Spur_5.0_rna.fna). Expression matrices were further analyzed using the R package Seurat v4.0.1 [37]. Empty droplets were filtered out based on the inflection point. Cells with at least 200 features and transcripts that were expressed in more than 3 cells were used for creating Seurat objects. Individual datasets were normalized by scaling gene expression with SCTtransform function. The top 2000 highly variable genes across the datasets were then used to integrate the datasets. Clustering analysis for visualization of the integrated data was conducted using the parameter dimensions = 40 and resolution = 0.6 based on UMAP projections. The replicates of HB, MB, EG, and LG stages were integrated with the Seurat IntegrateData function. Markers of clusters were identified with allMarkers() function using the MAST test and are presented in Supplementary Table S1. The correspondence between the transcript names from the genomic annotation and the more commonly used gene names in the literature is provided in Supplementary Table S2. Every embryo stage was analyzed separately, and cell clusters were classified based on the matching of the clusters’ markers and the existing experimental evidence on spatial and stage-specific expression.

### 2.6. Code Availability

The computer code used to generate the results presented in this manuscript is available at https://github.com/larisaok/Repeats_in_sea_urchin_genome, accessed on 30 September 2021.

## 3. Results

### 3.1. Unknown REs Contribute the Largest Portion to the S. purpuratus Genome REs

**Figure 1:**
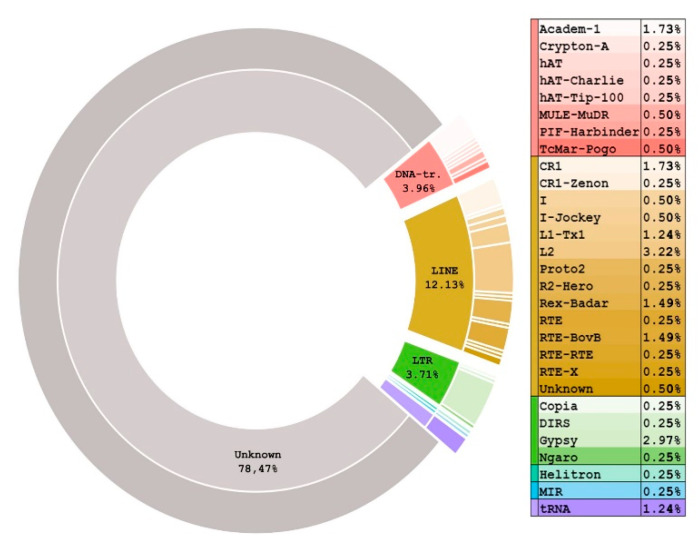
Donut plot of repetitive sequences in the genome of *Strongylocentrotus purpuratus,* version 5.0. The inner circle corresponds to the type of REs, the outer to the REs family.

In this study, we employed standard RepeatModeler + RepeatMasker pipeline to reveal all the repetitive sequences in the Spur 5.0 genome version. Although its assembly was not remotely perfect, we were able to identify many of the long-range sequences, such as *Helitron* transposons. Repeats in total occupied 33.75% of the *S. purpuratus* genome. The most prevalent were the unknown elements, which in total contributed 78.5% of repeats (27.35% of the total genome occupancy) (Figure 1). The second class, in terms of abundance, was the LINE elements (12.13% of the repeat occupancy and 4.32% in total), followed by the DNA-transposons (3.96% and 0.84% in total). The most abundant repeat in the genome by the genome portion was the *rnd-5/2144*—it occupied 0.44% of the genome with 8501 copies. Nevertheless, *rnd-1/78* outperformed it by copy number—16,282 copies; however, they occupied only 0.25% of the genome (see Supplementary Table S1). Interestingly, we found no *DNA/PiggyBac transposons* in Spur 5.0, while on the RepeatMasker site it was shown to occupy 4.2% of the genome. We checked this with the BLAST search of the *PiggyBac* consensus sequence in the *S.purpuratus* genome and found no occurrences. This may indicate that the resulting repeat repertoire may change from assembly to assembly.

**Figure 2:**
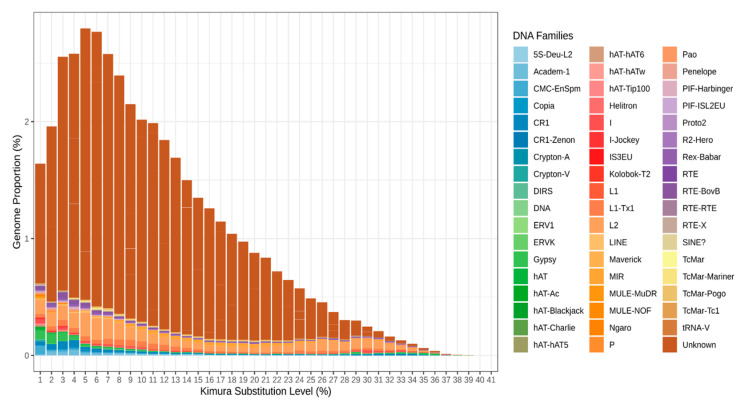
Interspersed repeat landscape in the *Strongylocentrotus purpuratus* genome. TE classes are marked with colors: SINEs, blue; LINE, red; LTR TE, yellow; DNA transposons, green; non-annotated repeats, brown.

The Kimura plot depicted in Figure 2 allows us to make some assumptions about the evolution of the repeats during *S. purpuratus* species origination. As can be clearly seen, the maximum genome portion was shifted and laid at 5–6% of Kimura substitution level.

As previously unknown repeats occupy most of the genome of sea urchin, their portion in any of the bars would be the largest one. From the shape of the bar plot, we can conclude that the majority of the repeats do not actively replicate in the genome. If the TEs were actively jumping, the distribution peak would shift to the left. In this species, it is highly likely that repeats have undergone active expansion in the near past. This assumption is correct for the Class II TEs and for those unknown. In contrast, DNA transposons have maximum abundance at the 1% level, and this can indicate that they are expanding in modern *S. purpuratus.*

### 3.2. RE Expression Significantly Changes during the Gastrulation Process

**Figure 3:**
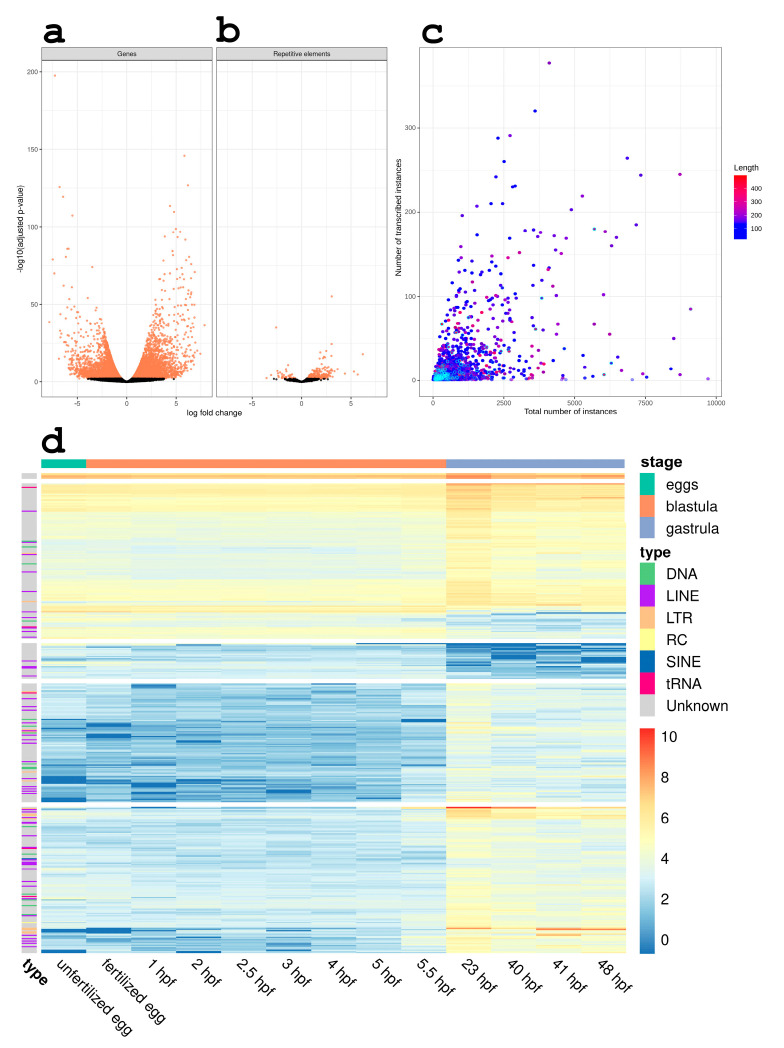
RE expression significantly changes during the gastrulation process irrespective of their origin and number of copies. Volcano plot showing differential expression of gene-coding transcripts (**a**) and RE (**b**) between blastula and gastrula stages. Each transcript is shown as a single point. Those with significantly changed levels are orange; black points are those without significant changes in transcription. (**c**) Scatter plot of expressed repetitive elements. X-axis represents the copy number of a given RE in the genome. Y-axis represents the number of transcribed copies of this element. Color gradient from blue to red indicates the length of the element. RE families with the length > 500 bp are shown with cyan dots. (**d**) Heat map of repetitive elements transcription that are differentially expressed during the gastrulation by embryo stages. Type column denotes the RE type. Class row denotes the embryo stage. Top 500 elements by their logFC were selected for this plot.

Analysis of the bulk RNA-seq data revealed that 404 REs significantly changed their expression at the gastrulation stage. It is worth noting that the number of upregulated REs (344) was significantly higher than the downregulated REs (60) (Figure 3b). Here, we evaluated the expression of all RE copies simultaneously without evaluating the locus-specific expression of the REs. Along with this, changes in gene expression were assessed (Figure 3a). The number of upregulated genes was 4266, for downregulated genes this was 5257. Clustering analysis revealed no group dynamics in RE or gene expression (Figure S1).

Next, we assessed the pattern of RE expression. As can be seen in Figure 3d, the expression pattern of RE remained consistent from the unfertilized egg to the pre-hatched blastula (approximately 5.5 hpf, dependent on water temperature) and changed greatly after this time point. We did not observe RE clustering according to family or class.

Subsequently, we evaluated the association of expression rate with the copy number of RE in the sea urchin genome with TEcandidates. We pooled together all the available stages in RNA-seq data and extracted RE instances that were being expressed in at least one of the assayed stages. The scatter plot illustrating the connection between the copy number of the RE in the genome and the number of their transcribing copies (Figure 3c) shows that there was a weak interaction between these values (the value of the Kendall correlation coefficient was 0.578, *z*-value = 160.98, *p*-value < 2.2 × 10^−16^). Furthermore, there was no correlation between the sequence length of the RE and copy number (the value of the Kendall correlation coefficient was 0.031, *z*-value = 8.58 *p*-value < 2.2 × 10^−16^). Taken together, this result may indicate that there are additional mechanisms controlling high-copy RE transcription.

### 3.3. REs Serve as Markers of Cell Populations According to scRNA-seq Data

#### 3.3.1. 64-Cell Stage

**Figure 4:**
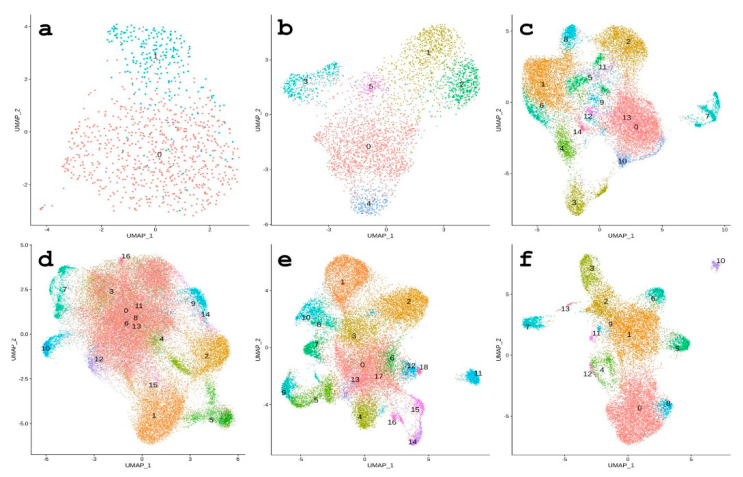
Results of the single-cell RNA-seq analysis. Umap plots of each embryo stage separately. (**a**) 64-cell embryo; (**b**) morula stage; (**c**) hatched blastula; (**d**) mesenchyme blastula; (**e**) early gastrula; (**f**) late gastrula.

**Figure 5:**
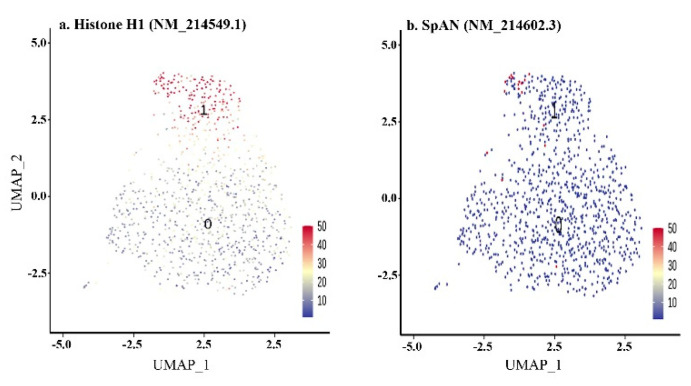
Feature plot of 64—cell stage. Cluster 0 corresponds to the vegetal pole of the embryo, cluster 1 to the vegetal pole. Each point corresponds to the single cell, and its color marks the level of either *H1* (**a**) or *SpAN* (**b**) gene expression.

An embryo reached the number of 60 cells at approximately 5 h post fertilization, depending on the water temperature. The cell cycle duration was around 30 min and the blastomere divisions were still synchronized. However, there was clear segregation between the animal and vegetal hemispheres. Therefore, there were two distinguishable clusters at the 64-cell stage (Figure 4a). Cluster 1 was highly likely to be the animal pole of the embryo based on marker expression—*Histone H1* (Figure 5a, Supplementary Table S1). The animal pole undergoes more intense cell divisions than a vegetal one, that is why histones marked this population. Several cells expressing the *SpAN* gene, known to be the marker of animal–vegetal polarity, could also be observed in cluster 1 (Figure 5b).

#### 3.3.2. Morula Stage

**Figure 6:**
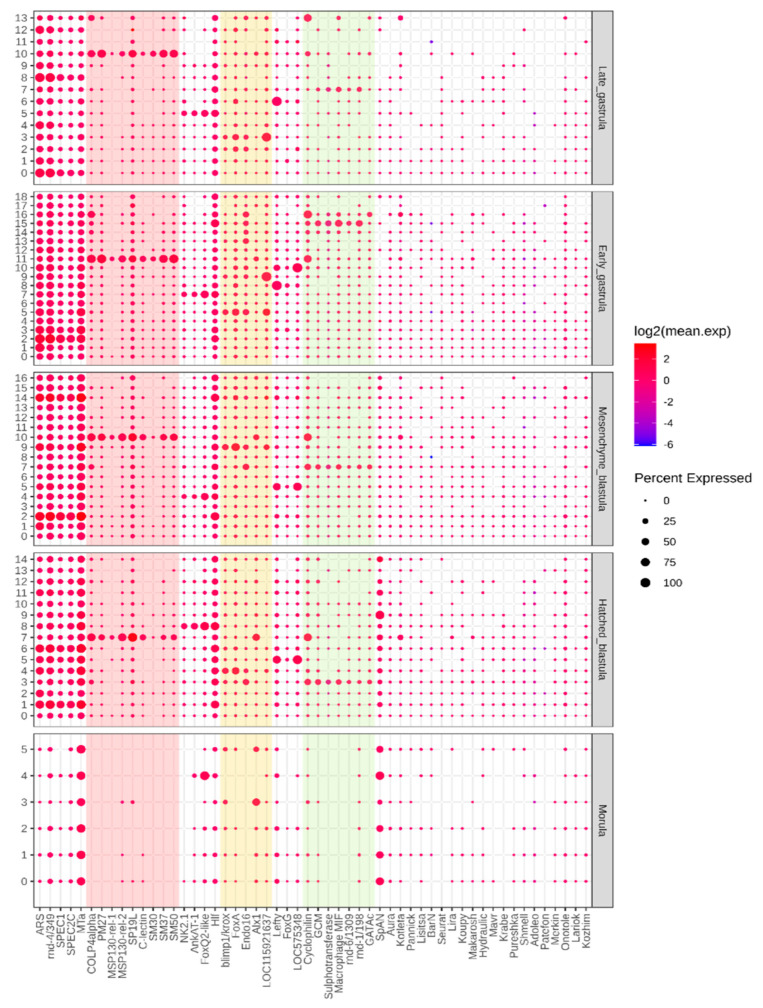
Results of the single-cell RNA-seq analysis. Dot plot of the expression of cell populations markers and REs. From left to right: ARS to MTa—aboral ectoderm markers; *COLP4alpha* to *SM50*—PMC markers (marked in pink); *NK2.1* to *Hlf*—apical plate markers; *blimp1*/*krox* to *LOC115921637*—endoderm markers; Lefty to *LOC575348*—oral ectoderm markers; *Cyclophilin* to *GATAc*—markers of SMC; *Aura* to *Kozhim*—novel REs.

Depending on the water temperature, there could be a morula stage immediately before gastrula formation. The number of clusters increased to six compared with the 64-cell stage (Figure 4b). Then, the large micromeres population in the 3rd cluster could be distinguished by the largest portion of Alx1 expressing cells. It could also be indirectly confirmed by the lowest portion of cells expressing *SpAN*, and this fact highlights its proximity to the vegetal pole. Moreover, this cluster demonstrated the largest portion of *SP19L* expressed cells and showed the expression of primary mesenchyme specific protein *MSP130-related-2* (*MSP130-rel-2*) (Figure 6, Supplementary Table S1).

The 5th cluster was highly likely to have originated from the endomesoderm cell lineage because of the specific marker, which marks the secondary mesenchyme domain (Supplementary Table S1, Supplementary Figure S2). There were also mesenchyme Alx1 and endoderm-specific FoxA genes, and their joint expression may be evidence of the intermediate expression pattern.

The remaining clusters could not be defined because there were no confident cluster markers. The pattern of the novel RE shown in the dot plot (Figure 6) revealed that most of the shown RE were expressed in all the clusters in the small portion of cells (0–25%). At the same time, there were several REs with cluster-specific expressions (Table 1). For example, *Lisitsa* and hydraulic elements were expressed in all clusters except for the 3rd, and *Lariok* RE was not expressed in the 5th cluster. Named RE sequences were queried against the RNAcentral database, and they were shown to match several previously described sea urchin lncRNAs sequences (Supplementary Table S3).

#### 3.3.3. From Hatched Blastula to Late Gastrula Stages

Since the hatched blastula stage, several embryo domains could be traced down—primary mesenchyme cells, secondary mesenchyme cells, aboral ectoderm, oral ectoderm, apical plate cells, and endoderm (Table 1). Cluster markers were annotated using Uniprot (Supplementary Table S4). These developmental domains could be distinguished at every assayed stage. There was also the compact *SFE1*-positive population of primordial germ cells.

#### 3.3.4. Aboral Ectoderm Domain

Aboral ectoderm forms the squamous mineralized epithelium covering the late embryo and larvae. This domain was represented at the HB stage through cluster 1 (Figure 4c). The expression of *SPEC 1A* gene and the isoform of *SPEC 2C*, both of which participate in mineralization, indicated the function of this cell population. However, there were no REs found as the confident markers of this cluster. At the MB stage, this domain was split into clusters 1 and 2 (Figure 4d). The 1st cluster expressed the *ARS1* gene as the marker. This gene encodes the arylsulfatase enzyme, known to be expressed exclusively in aboral ectoderm cells and their precursors. The 2nd cluster could be ascribed to the same population by the expression of *SPEC1A* and *SPEC2C*. This assumption could also be supported by *metallothionein A (MTa*) expression, which has a tropism to ectoderm. Both of these clusters had the rnd-4/349 element as the marker; however, in cluster 1, it is the negative marker, while in the 2nd cluster, it is the positive one (Figure 5, left white panel, Supplementary Table S1).

Similar to the MB stage, at the EG stage the 1st and 2nd clusters also represented the aboral ectoderm lineage (Figure 4e). *ARS1* assigned as a marker both clusters to this lineage. Interestingly, the 1st cluster had no RE as significant markers. The 2nd cluster expressed the *SPEC1A* gene and the rnd-4/349 element as the positive marker. This element was not detected either as the positive or as the negative marker in the 1st cluster, but there were two instances of this RE among the 2nd cluster markers.

The aboral ectoderm population in the LG was also split into 2 clusters: cluster 0 and cluster 8 (Figure 4f). Both of these clusters had the *ARS* gene among the significant markers, but only cluster 0 included two other genes—*SPEC1* and *XM_030980845.1* transcript—which was highly similar to *SPEC 2A*. Interestingly, both of these clusters continued to express the *rnd-4/349* RE. This RE, however, was ubiquitously expressed, but only at clusters 0 and 8 was it expressed by 100% of cells. Clusters 0 and 8 had four and six upregulated REs among the markers, respectively (Table 1).

#### 3.3.5. Apical Plate

The presence of the apical organ is a shared feature among the wide range of marine invertebrate larvae. This structure develops within a thickened epithelium at the animal pole of the larval body, termed the apical plate, and is often associated with a bundle of elongated cilia, the apical tuft. The apical organ of the sea urchin is composed of two clusters of nerve cells whose axons spread laterally across the anterodorsal edge of the oral hood, innervating the ciliated band [38,39]. This developmental domain begins to be distinguishable following the hatched blastula stage [40].

At the HB stage, the 8th cluster highly likely belonged to the apical plate, and this could be confirmed by the *AnkAT*-*1* and *Hlf* as the significant markers. This cluster also had the largest portion of the cells with *NK2.1* gene expressed. At this stage, this population only had one RE as the marker (Supplementary Table S1).

At the MB stage, the apical plate population was represented by the 4th cluster based on the expression pattern of *AnkAT-1* and *NK2.1* and having a ~25% portion of expressing cells in this cluster. It was the largest portion of all clusters. This cluster had four significant positive markers in total, and two of these were the transcripts that have a 90% identity to the *FoxQ2* protein, supporting this assumption [41]. This cluster had no RE as the significant marker (Table 1, Supplementary Table S1).

At the next assayed stages, EG and LG, this population was represented by the 7th and 5th clusters, respectively (Figure 4e, Figure 4f). This population had the same pattern of markers as the MB stage. However, at the EG stage, we observed increased diversity of RE among markers in this cluster. It also contained five REs among the significant markers list—three downregulated and two upregulated. At the LG stage, this population had *Hlf*, *Tektin*, and fibrous sheath-interacting protein 1 as the markers. This cluster had six REs as cluster markers and only one of them was negative (Table 1, Supplementary Table S1).

#### 3.3.6. Endoderm Lineage

The endoderm cell lineage begins to be morphologically distinguishable after the start of archenteron invagination. Nevertheless, this cell population starts to transcriptionally segregate much earlier [42]. In our study, this population was represented by the 4th cluster on the HB stage (Figure 4c). This assumption is based on the presence of *FoxA* and *Blimp*/*krox1* among the markers. In addition, there were the largest portions (~25%) of *FoxA* and Endo16 expressed cells. Both of these genes were the specific markers of the endoderm (Table 1, Supplementary Table S1).

At the MB stage, the endoderm lineage was likely represented by the 9th cluster (Figure 4d). This could be directly confirmed by the presence of the *FoxA* transcript in the markers list. It had also been transcribed in ~75% of cells of this cluster. Endo16, another marker of endoderm, had a ~25% portion of the cells expressing it in this cluster. Although its portion was not very high, the only value larger was the expression in the 7th cluster. There were no REs in the specific markers list of this cell population (Table 1, Supplementary Table S1).

The 5th cluster was likely to represent endoderm lineage in the EG due to having the largest portions of the cells with *FoxA* and *Endo16* expressed genes. Interestingly, this cluster had *FoxA* among the positive markers, while *Endo16* was the negative one. Remarkably, this cluster had no upregulated RE among the markers, but 10 downregulated ones.

In the LG stage, the 2nd and 3rd clusters represented endoderm cell population. Both of them had *FoxA* gene as the confident marker. In addition, these clusters had a high portion of *Endo16* (25–50%). Taken together, these facts exclude the oral ectoderm as the affiliation for this population in favor of endoderm. This population had a few REs among the markers—two positive and one negative, and four positive and two negative in the 2nd and 3rd clusters, respectively (Table 1, Supplementary Table S1).

#### 3.3.7. Primary Mesenchyme Cells

The first cell population that ingresses the blastocoel is the primary mesenchyme cell population (PMC). They form the skeletal spicules of the larvae. The ingression starts at the hatched blastula stage, and these cells are shown to originate from the large micromere cells.

At the HB stage, cluster 7 represented the PMC with ~75% of its cells expressing several specific markers: *COLP4alpha, Alx1, C-lectin protein, MSP130-2, SM37, and SM50*. Approximately ~50% of this cluster expressed *MSP130-1* and *P16*. A small percent of cells also expressed SM30. All of these genes were defined as confident markers. Furthermore, this cluster comprised seven REs as the positive markers and nine as the negative. The largest portion of the cells of this cluster expressed the Kotleta element (~25%).

At the MB stage, the 10th cluster obviously represented the PMC (Figure 4d). It had a similar pattern of expressed transcripts to the previous stage. There was also cyclophilin 1 in the significant markers list. There were 18 REs among cluster markers, four of them were upregulated.

Compared with this population at the previous embryo stage, the 11th cluster in EG had more spicule-specific genes among the markers. The number of REs among significant markers also dramatically increased—13 downregulated REs and 24 upregulated REs.

Although the transcription of genes was similar to the EG stage, the repertoire of the RE expression dramatically shrunk—only six REs were upregulated and one was downregulated in the 10th cluster of the LG stage (Table 1, Supplementary Table S1).

#### 3.3.8. Secondary Mesenchyme Cells

At the latest stage of archenteron invagination, the secondary mesenchyme cells (SMC) form the tip of the archenteron and provide the contact of the archenteron and inner blastocoel wall [42].

At the HB stage, it is highly likely cluster 3 represented the precursors of the SMC (Figure 4c). This assumption is based on the expression of GCM and GATAc genes serving as markers. Their expression took place specifically in non-skeletogenic mesoderm giving rise to pigment cells and immunocytes. The expression of cyclophilin indirectly supports this assumption, although it was expressed specifically in skeletogenic territories, but is also likely to be a marker of both primary and secondary mesenchyme cells [43]. Interestingly, the RE *rnd-6/1309* served as a marker of this cluster and was expressed in the largest portion of cells when compared with other clusters.

The SMC population at the MB stage was represented by the 7th cluster. Although this could not be confidently confirmed due to absence of specific markers, it was similar to the 4th cluster’s expression pattern of Endo16 and *Col4Alpha*. The combination of these markers suggests an endomesoderm cell population, which is most likely to be the SMC. There were also three upregulated REs in this cluster (Table 1, Supplementary Table S1).

At the EG stage, the 15th and 16th clusters belonged to the secondary mesenchyme domain. The 15th cluster population also had a ~25% portion of cells expressing *ENDO16* (Figure 5). Moreover, this cluster had the highest portions of *rnd-6/1309, rnd-1/198,* and *rnd-6/910* expressing cells compared to all other clusters, similarly to the previous stages. This cluster had the highest number of REs among all the clusters on this embryo stage—there were 53 of them in total, and 19 REs were upregulated. The 16th cluster had the Endo16 gene among the significant markers, but the portion of cells expressing this was not the highest of all clusters. The more specific marker was the GATAc gene, which is expressed in non-skeletogenic mesoderm, giving rise to pigment cells and immune cells. Interestingly, there were several significant actin isoforms in the markers list, and this fact may indicate that this population undergoes intensive cell migration, requiring actin to be highly expressed. Moreover, *cyclophilin 1* was also this population’s marker. This cluster contained 19 REs in total and six of them were positive (Table 1, Supplementary Table S1).

The presence of GCM in the markers list of the 7th cluster suggests that it belonged to the SMC domain at the LG stage. There were eight REs in the significant markers and six of them were positive. The patterns of *rnd-6/1309, rnd-1/198,* and *rnd-6/910* expression were similar, but the portion of expressing cells decreased in comparison to the population in previous embryo stages (Figure 5).

#### 3.3.9. Oral Ectoderm

There were two cell lineages showing no RE among the cluster markers in most of the embryo stages. These lineages were oral ectoderm and *SFE1*-positive cell population. We ascribed the clusters to the oral ectoderm population based on the expression of the Lefty gene (Figure 5, Table 1). This population was clearly distinguished starting from the hatched blastula stage. This population had no significant markers on the HB stage; however, it showed close to 100% of the Lefty expressing cells in the 5th cluster. At the MB and EG stages, the oral ectoderm population corresponded to the 5th and 8th clusters, respectively. Both of these clusters had the Lefty gene among the markers and had high 75% and 100% portions of expressing cells in these clusters, respectively. At the LG stage, the oral ectoderm was represented in the 6th cluster (Supplementary Table S1). This stage was the only one that had six REs among the cluster markers in the oral ectoderm. It seemed to be the late burst of the expression of RE in oral ectoderm.

#### 3.3.10. *SFE1*-Positive Population

The *SFE1* gene is the constituent of the fertilization envelope preventing polyspermic fertilization. It was shown to be transcribed specifically in oocytes, and no SFE1 mRNA was detectable in embryos or in somatic cells of the ovary by the *in situ* hybridizations [44]. Therefore, in this single-cell RNA-seq study, it was detected at MB, EG, and LG stages in the 12th, 4th, and 4th clusters, respectively (Figure 5, Supplementary Table S1). At the MB and EG stages, *SFE1* was the only positive marker, but at the LG stage there were four markers, and one of them appeared to be an RE. However, its adjusted *p*-value was rather high and close to the threshold of significance (0.015).

## 4. Discussion

### 4.1. Gene Regulatory Networks (GRNs), Primary Mesenchyme Cells (PMC)

GRNs play the role of a conductor of molecular processes of cell differentiation and morphogenesis during embryogenesis [45,46]. They are characterized by a hierarchical structure and are strictly one-directional. Obviously, the localization and topology of the GRN’s components are strictly defined in time and space, which is a prerequisite for obtaining a healthy embryo. Most of the studied GRNs are based on protein-coding genes that encode transcription factors, components of signaling pathways, and effector genes. The latter are mainly markers of terminal cell and tissue differentiation.

Transcription of the zygotic genome in sea urchin embryos begins soon after egg fertilization [47,48]. According to our data, at the 64-cell stage after the 6th cleavage, all cells divided into two large populations: animal and aboral (Figure 5a). Embryonic polarization is the result of the initial asymmetric assignment of maternal transcription factors in the zygote, which triggered specific gene cascades. During embryonic cell cleavage, other transcription factors are sequentially activated. Thus, by the beginning of gastrulation, the sea urchin embryo contains at least 15 different cell types [49,50]. This is also confirmed by our estimates. However, based on the processing of scRNA-seq data, we were able to accurately identify 17 clusters at this stage of embryogenesis (Figure 5d).

Prior to the sequencing of the sea urchin genome, a preliminary endomesoderm GRN was assembled based on several transcription factors and signaling molecules [31]. A detailed description of this regulatory network became possible after sequencing and annotation of the sea urchin genome [39], as well as sequencing of the transcriptomes of the early embryogenesis stages [51]. These studies revealed the main components and molecular logic of the gene regulatory network involved in cell differentiation and morphogenesis [8,31,32,33,46,52].

The early embryonic development of the sea urchin is of particular interest as an in vivo model of the epithelial–mesenchymal transition (EMT), which is an evolutionary-conservative cellular program [53,54]. The main embryonic region of epithelial–mesenchymal transition in sea urchin embryos is the vegetative pole, where mesenchymal cell precursors are located [55]. Micromeres are formed at the 16-cell stage after the 4th cleavage [56]. Micromeres produce two cell types after unequal cleavage, which consist of four small micromeres in the vegetative pole region that produce germ cells and four large micromeres that produce primary mesenchymal cells (PMCs) [55].

In view of the widespread interest in the EMT mechanisms, GRN that controls the differentiation micromeres—PMCs—has been well documented [32,55]. Progress in understanding the molecular machinery of EMT is based on the detailed analysis of the transcription of several genes involved in the early specification of micromere-PMC and PMC differentiation, for example: *pmar1, alx1, ets1, hesC, tbr, dri, hnf6, and snail* [32,39,57,58,59].

As mentioned above, the GRN’s design is based on the generalization and analysis of protein-coding gene transcription data. However, for many organisms, including humans and other mammals, it is shown that noncoding RNAs are the major players in the gene transcription regulation system [60].

In the course of embryogenesis, transcription of the total genome occurs [5], which results in the transcription of a significant number of ncRNAs. The latter is directly involved in the regulation of embryogenesis programs. However, despite intensive studies of the transcriptional profiles and GRNs of the sea urchin *S.purpuratus* [33,61], the information on ncRNA transcription is almost absent, especially for the early stages of embryogenesis.

### 4.2. Repetitive Elements as the Precursor of Functional ncRNA

It has been shown that repetitive sequences of genome, include transposons, can serve as a source for noncoding RNAs in evolution [60].

The TE-derived miRNAs regulate numerous biological processes and are present in all bilaterian animals. According to the miRNA online database (miRBase.org), there are 70 sequences [62,63,64] in the sea urchin *S. purpuratus*, while 2588 and 1915 miRNA sequences were described in humans and mice, respectively [65]. It was shown that about 20% of human miRNAs are transcribed from the TE sequences [66]. No such information is available for the sea urchin. Researchers have previously shown the critical importance of miRNAs for early embryogenesis of the sea urchin [64], but there is a lack of detailed information in the literature about possible precursors of these regulatory molecules. In our study, we also focused our attention on another class of ncRNAs.

The TE–derived long non-coding RNA in sea urchin, LncRNA and lincRNA (The Long Intergenic Noncoding RNA, one class of lncRNA), are involved in many cellular processes [67,68], including the epigenetic regulation [69,70,71] and embryogenesis [72] encompassing transcriptional silencing, cellular reprogramming, and X-chromosome inactivation.

LncRNAs are less conservative than miRNAs. It has been shown that most of the vertebrate lncRNAs are sufficiently specific only within taxon. This can be explained by their variability and fairly rapid evolution [73]. It was shown that about 100 new lncRNAs appear in primates over one million years. Such a rate exceeds the rate of appearance of new protein-coding genes. According to the study, the lncRNA expression pattern also changes faster than the expression of protein-coding genes [74,75].

About one thousand lncRNAs described for humans have homologues in other mammals, but only hundreds of lncRNAs have homologues outside the taxon [73]. Humans and sea urchins had a common ancestor more than 500 million years ago, so there are almost no lncRNAs similar in structure and function at this time [30,73]. However, the absence of homologues may be due to the lack of a systematic approach in the study of sea urchin ncRNAs.

Most lncRNAs, including lincRNA, contain TE sequences [60]. According to the RIDL theory, TE exon sequences act as functional domains in lncRNAs [76]. It is known that TE sequences are found in 50% or more of the lncRNAs that have been studied, but there are significant species differences [77,78]. The study conducted on humans and mice showed that the contribution of different TE families to lncRNAs globally reflects the amount of each family in the genome, except for the LINE depletion in exons and promoters of lncRNAs. Some TE-derived domains in lncRNAs have been shown to be functionally important—they form RNA\DNA\protein-binding domains [76]. For example, LINE2 and MIR elements contribute to the nuclear enrichment of lncrnA, which enables them to modulate the gene expression [78].

The new repetitive sequences described in our study range in size from 75 to 494 bp, but, at the same time, they are not independent transcripts and are parts of longer molecules in the form of a domain (see Supplementary Materials Table S2). All of these sequences have high transcriptional activity in the cluster, which we identified as PMC. At this point, we can assume that their functional activity is somehow related to PMC differentiation, and they are involved in either silencing certain gene clusters or, conversely, in activating silent genes that are necessary for the implementation of this differentiation program.

Since the gene regulatory network responsible for the differentiation of primary mesenchymal cells has been described in sufficient detail, we can assume that a systematic overlay of the regulation of the transcriptional level of these lncRNAs will provide insight and a new perspective on the combined effect of transcriptional and post-transcriptional regulation during the isolation of the PMC population.

### 4.3. scRNA-Seq Results

In the source paper, 22 cell types were identified in the merged dataset at the gastrula stage; this corresponds well to the established fate maps of urchin embryos [35,42,50]. The original pipelines used Cell Ranger, which considers only protein-coding transcripts. By adding RE-derived transcripts, we increased the number of features and, consequently, the resolving power of the method. However, we were not able to assign cell populations for all clusters due to the lack of information about many urchin genes.

It turned out that the expression of RE can be specific to cell populations and persists between stages. If they expressed transcriptional noise, they would not be defined approximately anywhere as cluster markers [37]. However, our approach also had limitations because we used 10x Genomics Single-cell v3 chemistry for single cells, which is focused on poly-A RNA capture. Some types of ncDNA are transcribed with other polymerases and so they are lost. It would therefore be interesting to show whether the pattern of RE could be preserved using a different chemistry.

Nevertheless, thanks to our approach we were able to show in principle that REs are important in embryonic development and, on a par with protein-coding transcripts, can determine the fate of cells in embryogenesis.

As forspecific features, different numbers of REs among the markers were also found depending on the population and the stage of embryonic development. The most interesting phenomenon in this case was the population of mesenchymal cells—they had the highest number of REs. There can be two opposite explanations for this.

The first explanation is that these are terminally differentiated cells, which means that there is no need to preserve multipotency, and the mechanisms involved in suppressing transcription do not work. This is supported by the existence of mechanisms to suppress TE activity, which are particularly active in germline cells.

This idea is opposed by another explanation, suggesting that in the cells of this population, on the contrary, there is a modulation of transcription due to these REs. For example, their transcription is associated with some regulatory elements or, on the contrary, with the suppression of gene transcription. It may also happen that one copy of the RE is expressed suppressing the others. Meanwhile, this hypothesis can only be tested experimentally, which was not provided within the framework of this paper.

### 4.4. Repeats as the Putative Evolutionary Force

As a result of the RepeatModeler + RepeatMasker pipeline run, we found that 44% of the urchin genome was occupied by repeats, and most of them (78.74%) belonged to previously unknown families. Currently, the classification of RE is too complicated due to the limited number of well-assembled genomes. The DFAM consortium continuously develops new approaches to classification and updates consensus sequences of REs. Our result agrees in general with the result of the authors of the latest *S. purpuratus* 5.0 genome assembly. Using the alternative WindowMasker pipeline, they masked 39% of the genome. It is noteworthy that a masked portion in the genome is not a good subject to compare between different taxa because the percentage of ncDNA varies greatly even between neighboring species [79]

RE expansion is a phenomenon that can work as an evolutionary factor. Without control, REs can expand and disrupt gene functions. On the other hand, organisms have learned both to resist expansion (e.g., piRNA in *Drosophila*) and to “tame” such repeats (e.g., transposons in *Drosophila* telomeres, transposon origin of segment recombination in adaptive immunity). In our study, we revealed the co-option of the REs in embryo development. According to our assumptions based on the analysis of the RE repertoire, we can say that a massive expansion of REs occurred in sea urchin in the recent evolutionary past. Perhaps this continues today within a narrow group of DNA transposons. It is worth noting that a careful classification of the other families may shift the estimates of the timing of this expansion, but it is unlikely to dramatically change the overall picture. Summarizing the resulting data, we can say that it is possible that further investigations will reveal new mechanisms of REs in the genome and modulation of transcription.

## 5. Conclusions

Four major conclusions can be drawn from the present study: (1) REs significantly change their expression during the gastrulation process; (2) the expression of an RE is independent of the number of its copies in the genome; (3) RE expression distinguishes cell lines from the morula stage (4) expression of RE is observed in all cell lineages and they are detected as population markers at a single-cell resolution level. The pattern of RE expression can vary between cells of different lineages.

## Figures and Tables

**Table 1:** Cell lineages in stages from hatch blastula to late gastrula and their marker genes and REs.

Layer	Lineage	Stage	Clusters	Marker Genes	Number of Marker RE	
					Upregulated	Downregulated
Ectoderm	Aboral ectoderm	HB	1	*Spec1A, Spec2C, MTa*	1	0
		MB	1, 2	*Spec1A, Spec2C, MTa, ARS*	1	3
		EG	1, 2	*Spec1A, Spec2C, ARS*	2	1
		LG	0, 8	*Spec1A, Spec2C, ARS*	10	0
	Apical plate	HB	8	*Hlf, AnkAT-1, FoxQ2-like*	1	6
		MB	4	*FoxQ2-like*	0	0
		EG	7	*FoxQ2-like, AnkAT-1*	2	3
		LG	5	*Hlf, AnkAT-1, NK2.1, FoxQ2-like*	5	1
	Oral ectoderm	HB	5	*Lefty*	0	0
		MB	5	*Lefty*	0	0
		EG	8	*Lefty*	0	0
		LG	6	*Lefty, FoxA*	6	3
Endoderm	Endoderm	HB	4	*FoxA, blimp1/krox*	0	0
		MB	9	*FoxA*	0	7
		EG	5	*FoxA*	0	10
		LG	2, 3	*FoxA*	6	3
Mesoderm	PMC	HB	7	*MSP130-rel-2, P19, Col4alpha, SM37, Alx1, Cyclophilin, PM27, C-lectin-like, SM37*	7	9
		MB	10	*MSP130-rel-2, P19, Col4alpha, SM37, Alx1, Cyclophilin, PM27, C-lectin-like, SM37*	4	14
		EG	11	*MSP130-rel-2, P19, Col4alpha, SM37, Alx1, Cyclophilin, PM27, C-lectin-like, SM37, SM29, SM50*	24	13
		LG	10	*MSP130-rel-2, P19, Col4alpha, SM37, Alx1, Cyclophilin, PM27, C-lectin-like, SM37, SM29, SM50*	6	1
	SMC	HB	3	*gcm, GATAc, cyclophilin*	6	1
		MB	7	*Sulphotransferase*	3	15
		EG	15, 16	*Sulphotransferase, gcm, endo16, GATAc*	25	47
		LG	7	*Sulphotransferase, gcm, endo16, GATAc, Macrophage MIF*	6	2
	*SFE1*-positive	HB	-	*-*	-	-
		MB	12	*SFE1*	0	0
		EG	4	*SFE1*	0	0
		LG	4	*SFE1*	1	0

## Data Availability

Publicly available transcriptome data from *S.purpuratus* embryos were downloaded from the SRA database under accession number PRJNA531297. The data contain RNA-sequencing reads of unfertilized eggs, embryos from 2, 4, 8, 18, 32, 64, and 128 cell stage (blastula stage) and 24, 40, 41, and 48 h after fertilization (gastrula stage), which were maintained at 15 °C. Raw reads of single-cell RNA-sequencing data were downloaded from the SRA database stored under accession number GSE149221 (Foster, 2021). Eight developmental stages in *S.purpuratus*, from the eight-cell stage to late gastrula stage.

## Supplementary Materials

The following are available online at https://www.mdpi.com/article/10.3390/biomedicines9111736/s1.
