# Supplementary material for: Pattern of Repetitive Element Transcription Segregate Cell Lineages during the Embryogenesis of Sea Urchin Strongylocentrotus purpuratus
Source: Biomedicines. 2021 Nov 21;9(11):1736. doi: 10.3390/biomedicines9111736 (PMC8615465; doi:10.3390/biomedicines9111736)
Supplement: Supplementary file 1 [file biomedicines-09-01736-s001.zip › biomedicines-1425482-supplementary.pdf]

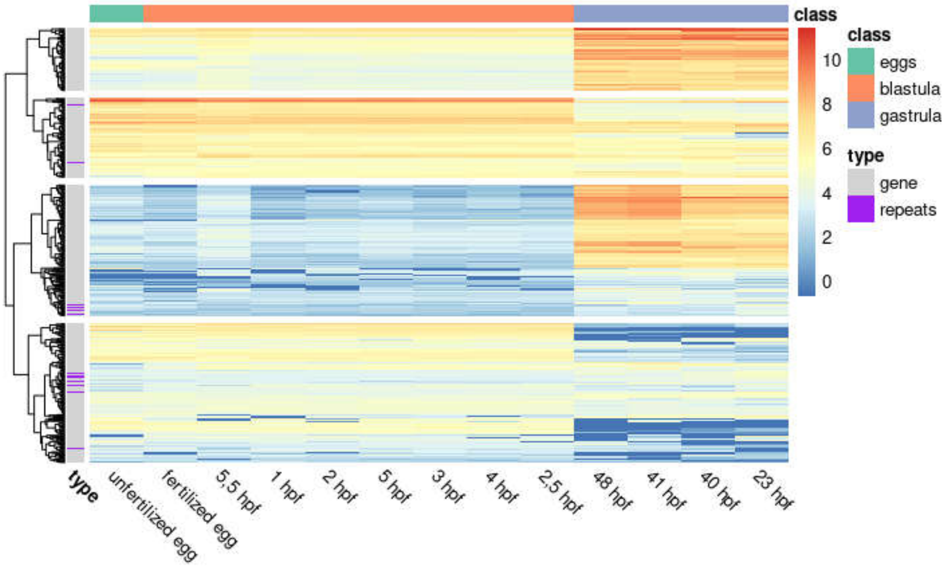

Figure S1. Heatmap of top 500 most changed transcripts by fold change. Type column denotes the type of a transcript - gene(grey) or repeat(magenta). Class row denotes the embryo stage.

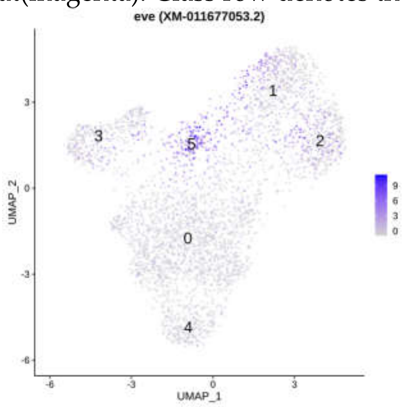

Figure S2. Feature plot of the expression of the eve gene on the morula stage of a sea urchin embryo. Color gradient from grey to blue indicates the scaled expression of the eve.

Supplementary table S1
https://docs.google.com/spreadsheets/d/1OqVv8m-fjiAbZYRZKACh8_xOCxLRlGebiuGpfrxeUsw/edit#gid=1851883386

Supplementary table S2
https://docs.google.com/spreadsheets/d/12LczlFZC09yAFI3s95KgHG8IVI_Piw8tMOmmPbf5zhg/edit#gid=0

Supplementary table S3
https://docs.google.com/spreadsheets/d/1sGarn5q2rXw5B8YhdboHdRZ6Tu7eHWGhMR8w2HxggQk/edit#gid=0

Supplementary table S4
https://docs.google.com/spreadsheets/d/1gLaxOW4OXtFQIwbXCXsK_ZpUkSFtaeEIeS1CMWiryTI/edit#gid=0
